# Dose–Response Association Between High-Density Lipoprotein Cholesterol and Stroke: A Systematic Review and Meta-Analysis of Prospective Cohort Studies

**DOI:** 10.5888/pcd18.200278

**Published:** 2021-05-13

**Authors:** Ranran Qie, Leilei Liu, Dongdong Zhang, Minghui Han, Bingyuan Wang, Yang Zhao, Dechen Liu, Chunmei Guo, Quanman Li, Qionggui Zhou, Gang Tian, Shengbing Huang, Xiaoyan Wu, Pei Qin, Jianxin Li, Jie Cao, Ming Zhang, Jianfeng Huang, Jie Lu, Dongsheng Hu

**Affiliations:** 1 Department of Epidemiology and Health Statistics, College of Public Health, Zhengzhou University, Zhengzhou, Henan, People’s Republic of China; 2 School of Public Health, Shenzhen University Health Science Center, Shenzhen, Guangdong, People’s Republic of China; 3 Study Team of Shenzhen’s Sanming Project, The Affiliated Luohu Hospital of Shenzhen University Health Science Center, Shenzhen, Guangdong, People’s Republic of China; 4 Department of Epidemiology, Fuwai Hospital, National Center for Cardiovascular Diseases, Chinese Academy of Medical Sciences and Peking Union Medical College, Beijing, People’s Republic of China

## Abstract

**Introduction:**

Studies investigating the effect of high-density lipoprotein cholesterol (HDL-C) on stroke and stroke subtypes have reached inconsistent conclusions. The purpose of our study was to clarify the dose–response association between HDL-C level and risk of total stroke and stroke subtypes by a systematic review and meta-analysis.

**Methods:**

We performed a systematic search of PubMed, Embase, and Web of Science databases through July 30, 2020, for prospective cohort studies that reported the HDL-C–stroke association and extracted the estimate that was adjusted for the greatest number of confounding factors. Restricted cubic splines were used to evaluate the linear and nonlinear dose–response associations.

**Results:**

We included 29 articles, which reported on 62 prospective cohort studies including 900,501 study participants and 25,678 with stroke. The summary relative risk per 1-mmol/L increase in HDL-C level for total stroke was 0.82 (95% CI, 0.76–0.89; *I* ^2^ = 42.9%; n = 18); ischemic stroke (IS), 0.75 (95% CI, 0.69–0.82; *I* ^2^ = 50.1%; n = 22); intracerebral hemorrhage (ICH), 1.21 (95% CI, 1.04–1.42; *I* ^2^ = 33.4%; n = 10); and subarachnoid hemorrhage (SAH), 0.98 (95% CI, 0.96–1.00; *I* ^2^ = 0%; n = 7). We found a linear inverse association between HDL-C level and risk of total stroke and SAH, a nonlinear inverse association for IS risk, but a linear positive association for ICH risk. The strength and the direction of the effect size estimate for total stroke, IS, ICH, and SAH remained stable for most subgroups. We found no publication bias with Begg’s test and Egger’s test for the association of HDL-C level with risk of total stroke, IS, and ICH.

**Conclusion:**

A high HDL-C level is associated with reduced risk of total stroke and IS and an increased risk of ICH.

**Summary**

**What is already known on this subject?**

Previous epidemiologic studies reported that HDL-C protected against the development of stroke. However, several recent cohort studies found a positive association between HDL-C level and intracerebral hemorrhage. Also, whether a dose–response association between HDL-C level and stroke subtypes exists remains unclear.

**What is added by this report?**

Our results showed an 18% reduction in the relative risk of total stroke and a 24% reduction for ischemic stroke, but a 21% increase in intracerebral hemorrhage per 1-mmol/L increase in HDL-C level.

**What are the implications for public health practice?**

Reasonable control of HDL-C level will prevent and control incident stroke. Our findings may facilitate the development and promotion of blood lipid prevention strategies aimed at reducing stroke risk.

## Introduction

Stroke is highly prevalent worldwide, and the number of people who experience stroke increased to more than 104.2 million in 2017 (1). From 1990 through 2017, the disability-adjusted life-years for stroke were about 132.0 million in 195 countries (2). Moreover, stroke is the second leading cause of death in the world, accounting for 6.2 million deaths globally in 2017. Of these deaths, about 2.7 million were due to ischemic stroke (IS), 3.0 million to intracerebral hemorrhage (ICH), and 0.5 million to subarachnoid hemorrhage (SAH) (3,4). However, much of the stroke burden could be prevented by managing and controlling modifiable risk factors.

Many prospective cohort studies reported that a high-density lipoprotein cholesterol (HDL-C) level protected against the development of stroke (5–11). However, the “good cholesterol” label for HDL-C has been challenged by several recent randomized controlled trials demonstrating that HDL-C–elevating therapy increased the risk of cardiovascular diseases (12,13). Thus, a full understanding of the effect of HDL-C level on stroke and stroke subtypes is warranted. Only one systematic review, conducted in 2008, examined the association between HDL-C level and risk of total stroke (14). Another meta-analysis in 2013 investigated the association between HDL-C level and risk of hemorrhagic stroke (15). However, up to 10 more cohort studies have been published recently on the association of HDL-C level with total stroke, ICH, and SAH, showing inconsistent results (9–11,16–24). No meta-analysis has been performed on the association of HDL-C level with IS, and a dose–response meta-analysis on the association of HDL-C level with total stroke and IS is lacking. We therefore performed this systematic review and dose–response meta-analysis of prospective cohort studies to quantitatively evaluate possible linear or nonlinear associations between baseline HDL-C level and risk of total stroke, IS, ICH, and SAH.

## Methods

### Data sources and searches

We followed the protocol for the Preferred Reporting Items for Systematic Reviews and Meta-Analyses (PRISMA) Statement for our meta-analysis (25). We conducted a systematic literature search of PubMed, Embase, and Web of Science databases for all reports of prospective cohort studies that examined the association between HDL-C level and stroke and were published through July 30, 2020, with no restriction on language. We also searched the reference lists of all related articles and reviews.

### Study selection

Two authors (R.Q. and M.H.) independently searched articles, selected relevant studies based on their title and abstract, then evaluated these articles by reviewing the full text. Inclusion criteria for prospective cohort studies were as follows: 1) study participants were aged ≥18 years; 2) the study investigated the association between HDL-C level and risk of stroke or stroke subtypes; 3) the study reported the effect estimates, relative risks (RRs), or hazard ratios (HRs), with 95% CIs for ≥3 HDL-C categories or per-unit increase in HDL-C level; and 4) the study reported the number of cases, exposed person-years, or participant numbers in each category of HDL-C level. We excluded cross-sectional and case-control studies, commentaries, letters, reviews, meta-analyses, and studies with unusable data. If data from the same study were reported more than once, only the most recent and complete data were included.

### Data extraction and quality assessment

R.Q. and L.L. independently extracted the following information from each study: first author, publication year, study name, study location, follow-up period, age range, sex, stroke and HDL-C assessment method, baseline levels of HDL-C, case number of per-category HDL-C exposure, total persons or person-years of per-category HDL-C exposure, reported RRs or HRs and 95% CIs for each HDL-C category, and adjusted covariates. Included studies were assessed for quality according to the 9-point Newcastle–Ottawa Quality Assessment Scale (NOS) (26). Any discrepancy was resolved by discussion with a senior investigator (D.H.).

We classified stroke, which included embolic infarction, large-artery occlusive infarction, lacunar infarction, and unclassified, as ICH, SAH, and IS (10). Some studies include all types of stroke for analysis and we call it total stroke in this meta-analysis. The lowest HDL-C category was the reference. For studies that did not choose the lowest category as the reference category, we reformulated RRs to set the lowest HDL-C category as the reference (27). When HDL-C levels were reported in milligrams per deciliter (mg/dL), we used the scaling factor of 38.67 to translate 1-mg/dL HDL-C to 1-mmol/L HDL-C. Studies that provided results separately for men and women or reported multiple stroke subtypes within an article were treated as independent studies. For studies reporting results separately for fatal and nonfatal stroke, we combined the RRs and then included the pooled RR in the meta-analysis.

### Data synthesis and analysis

We considered the RR and 95% CI of the effect size for all studies. The reported HRs in the primary studies were considered equal to RRs (28). We first used the DerSimonian and Laird random-effects model, which considers both within-study and between-study variation, to calculate summary RRs and 95% CIs for high versus low HDL-C level (29). Studies reporting only a continuous risk estimate of stroke were excluded from our analysis. We then pooled the study-specific dose–response RRs and 95% CIs per 1-mmol/L increase in HDL-C level (29).

We used generalized least squares regression to estimate the study-specific dose–response association (30). The natural RRs and CIs across categories of HDL-C level were used to compute study-specific slopes (linear trends) and 95% CIs. A generalized least squares regression model estimates the linear dose–response coefficients and considers the covariance for each exposure category within each study because they are estimated relative to a common referent HDL-C level category. In this method, the distribution of cases and person-years, or cases and noncases, with the RRs and estimates of uncertainty (eg, CIs) for ≥3 quantitative categories of exposure were required. If studies reported only the total number of cases or person-years, the number of person-years or cases in each category was obtained from the total number of person-years or cases divided by the number of reported categories. We assigned the mean, median, or midpoint of HDL-C level in each category to the corresponding risk estimate. When the lowest or highest categories were open-ended, we assumed the width of the category to be the same as the closest category when estimating the midpoint (31). For the studies already reporting a linear dose–response trend for per n-mmol/L increase in HDL-C level, we calculated the dose–response RRs per 1-mmol/L increase in HDL-C level with this formula: RR_1_ = EXP (LN (RR_n_)/n*1), where RR_1_ represents the dose–response RRs for each 1-mmol/L increase in HDL-C level and RR_n_ represents the dose–response RRs for each n-mmol/L increase in HDL-C level (EXP: exponential function; LN: log base e) (32). All study-specific dose–response RR estimates were then pooled by using the DerSimonian and Laird random effects model (29). With heterogeneity (*I* ^2^) ≥50%, a random-effects model was used to calculate the summary RRs and 95% CIs; otherwise a fixed-effects model was used, which considered both within- and between-study variation. The Hartung-Knapp-Sidik-Jonkman method was used to evaluate the stability of results for N <10 (33). A potential nonlinear association was examined by modeling HDL-C level by using restricted cubic splines with 3 knots located at the 25th, 50th, and 75th percentiles of the distribution (34). The *P* for nonlinearity was calculated by testing the null hypothesis that the coefficient of the second spline is equal to zero (35).

Heterogeneity was assessed by Cochran Q and *I* ^2^ statistics (36). For the Q statistic, *P* < .10 was considered significant. For the *I* ^2^ statistic, *I* ^2^ values of 0%, 25%, 50%, and 75% were considered to reflect no, low, moderate, and high heterogeneity, respectively. We also performed subgroup analyses by sex, region, follow-up period, publication year, sample size, and the covariates (alcohol drinking, education, body mass index, systolic blood pressure, physical activity, lipid-lowering medication use, and other lipid profile parameters) adjusted in the analysis.

A sensitivity analysis was performed to assess the influence of each individual study by omitting 1 study at a time and calculating a pooled estimate for the remainder of the studies (37). Potential publication bias was assessed with Egger’s and Begg’s tests (38,39). Conversion from DerSimonian-Laird results to Hartung-Knapp-Sidik-Jonkman results involved using Microsoft Excel software (Microsoft Corp). Other analyses were conducted with Stata 12.1 (Stata Corp), and all tests were 2-sided with a significance level of *P *< .05.

## Results

**Literature search and study characteristics**. Our literature search identified 7,366 articles; 1,113 were duplicates, leaving 6,253. After screening the titles and abstracts, we selected 201 potentially eligible articles. After detailed evaluation, we included 29 articles describing 62 prospective cohort studies in our meta-analysis with a total of 900,501 study participants of which 25,678 had stroke (5–11,16–24,40–52).

Eleven studies were conducted in Asia (including Iran and Israel) (7,8,10,17,18,20,21,23,24,46,52), 9 in the United States (9,19,22,40,42,44,48–50), 7 in Europe (5,11,16,41,43,47,51), and 2 in Australia (6,45). Three prospective cohorts included only men (5,51,52), another 3 included only women (8,40,49), and the rest included both sexes (Table 1). The mean NOS score was 8.24, which indicates the high quality of the articles included in the meta-analysis.

**Table 1:** Characteristics of Prospective Cohort Studies Reviewed, Dose–Response Association Between High-Density Lipoprotein Cholesterol and Stroke^a^

Study	Country	Year	Age, y (SD)^b^	Follow-up, y	Sample Size, N (% Men)	Main Outcomes	NOS^c^
Watanabe et al (23)	Japan	2020	55.0 (13.4)	10.7	11,027 (38.9)	Total stroke, IS, ICH, SAH	9
Zhang et al (22)	US	2019	52.7	17	36,030 (44.5)	Total stroke	8
Gu et al (24)	China	2019	50.4 (11.6)	6-19	267,500 (59.6)	IS	9
Rist et al (40)	US	2019	≥45	19.3	27,937 (0)	ICH, SAH	9
Liu et al (21)	China	2019	20–80	3.6	42,005 (61.9)	Total stroke, IS	8
Saito et al (10)	Japan	2017	40–69	15	30,736 (34.4)	Total stroke, IS, ICH, SAH	8
Anne et al (41)	Norway	2017	≥30	12.8	27,936 (47.4)	IS	9
Harandi et al (18)	Iran	2016	≥35	10	6,323 (NA)	Total stroke	8
Glasser et al (19)	US	2016	≥45	6.9	23,867 (45.0)	Total stroke, IS	8
Hirata et al (20)	Japan	2016	≥30	18	7,019 (42.0)	Fatal total stroke, fatal IS	9
Pikula et al (42)	US	2015	64 (10)	9	6,276 (44.0)	IS	8
Reina et al (9)	US	2015	45–84	9.5	6,814 (47.0)	Total stroke	8
Tohidi et al (17)	Iran	2013	≥50	9.1	2,620 (46.0)	Total stroke, IS	8
Zhang et al (11)	Finland	2012	25–74	20.1	58,235 (NA)	Total stroke, IS, ICH, SAH	8
Wieberdink et al (43)	Netherlands	2011	58.8–68.5	9.7	5,773 (NA)	ICH	9
Hamer et al (16)	England	2011	NA	NA	13,778 (NA)	Fatal total stroke	7
Simons et al (45)	Australia	2009	≥60	16	2,805 (44.0)	IS	8
Willey et al (44)	US	2009	68.8 (10.3)	7.5	2,940 (36.5)	IS	7
Noda et al (46)	Japan	2009	40–79	10	91,219 (33.8)	Fatal ICH	9
Holme et al (47)	Sweden	2009	30–85	11.8	148,600 (56.5)	IS	8
Sturgeon et al (48)	US	2007	≥45	13.5	21,680 (44.2)	ICH	7
Kurth et al (49)	US	2007	≥45	11	27,937 (0)	IS	9
Psaty et al (50)	US	2004	≥65	7.5	4,885 (40.0)	IS	8
Curb et al (8)	Japan	2004	71–93	6.3	2,444 (0)	Total stroke	9
Soyama et al (7)	Japan	2003	35–79	10	4,989 (30.5)	Total stroke	9
Simons et al (6)	Australia	2001	≥60	10.8	2,805 (44.0)	Total stroke	8
Wannamethee et al (5)	England	2000	40–59	16.8	7,735 (100)	Total stroke	9
Leppala et al (51)	Finland	1999	50–69	6	28,519 (100.0)	ICH, SAH	7
Tanne et al (52)	Israel	1997	≥42	21	8,586 (100.0)	Fatal IS	8

Abbreviations: ICH, intracerebral hemorrhage; IS, ischemic stroke; NA, not available; NOS, Newcastle–Ottawa Scale; SAH, subarachnoid hemorrhage.

a Based on a systematic search of publications on PubMed, Embase, and Web of Science databases through July 30, 2020.

b Some articles reported mean age and SDs of included participants, and other articles reported only age range.

c The Newcastle–Ottawa Scale (NOS) for assessing the quality of nonrandomized studies in meta-analysis (26).

**Figure F1:**
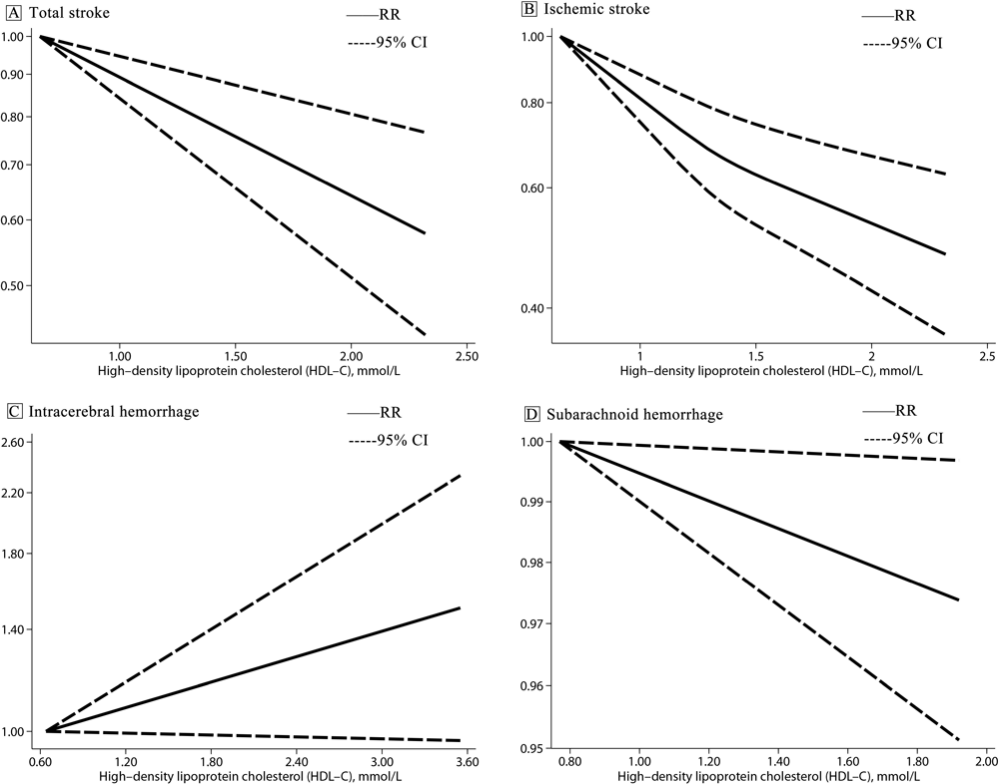
Linear dose–response association between high-density lipoprotein cholesterol and risk of stroke and stroke subtypes modeled with restricted cubic splines. Graph A shows total stroke; B, ischemic stroke; C, intracerebral hemorrhage; and D, subarachnoid hemorrhage.

**HDL-C level and risk of total stroke**. To explore the association between HDL-C level and risk of total stroke, we examined 18 studies that included 256,427 participants overall and 12,328 people with stroke. We excluded 8 studies in comparing the highest versus lowest category of HDL-C because they provided only a continuous risk estimate. The pooled RR was 0.79 (95% CI, 0.72–0.87; *I* ^2^ = 46.4%; *P*_heterogeneity_ = .05) (Table 2). The 18 studies were included in the dose–response analysis; the pooled RR for total stroke was 0.82 (95% CI, 0.76–0.89) per 1-mmol/L increase in HDL-C level, with low heterogeneity (*I* ^2^ = 42.9%; *P*_heterogeneity_ = .03) (Table 3)[Fig F1] . We found a linear dose–response association between HDL-C level and risk of total stroke (*P*_nonlinearity_ = .96) (Figure). No evidence of heterogeneity was detected between subgroups (Table 4). We observed an inverse association for most subgroups, except a nonsignificant association in studies of women, with a follow-up period of less than 10 years, without adjustment for physical activity or without adjustment for other lipid profile parameters (Table 4).

**Table 2:** Risk Of Stroke And Stroke Subtypes With Highest Versus Lowest High-Density Lipoprotein Cholesterol, Systematic Review and Meta-Analysis of Prospective Cohort Studies

Study (Reference Citation)	Sex	Study Year	Relative Risk (95% CI)	Weight (%)^a^
**Total stroke**				
Watanabe et al (23)	Men and women	2020	0.68 (0.49–0.95)	8.63
Zhang et al (22)	Men and women	2019	0.78 (0.63–0.96)	21.31
Saito et al (10)	Men	2017	0.78 (0.61–0.99)	16.12
Saito et al (10)	Women	2017	0.93 (0.73–1.17)	16.99
Hirata et al (20)	Men and women	2016	1.39 (0.67–2.89)	1.77
Zhang et al (11)	Men	2012	0.98 (0.75–1.27)	13.63
Zhang et al (11)	Women	2012	0.70 (0.53–0.93)	11.96
Curb et al (8)	Men	2000	0.37 (0.17–0.81)	1.55
Soyama et al (7)	Men and women	2003	0.35 (0.16–0.74)	1.61
Wannamethee et al (5)	Men	2000	0.68 (0.46–0.99)	6.44
Overall^b^	—	—	0.79 (0.72–9.87)	100.0
**Ischemic stroke**				
Watanabe et al (23)	Men and women	2020	0.75 (0.50–1.12)	4.89
Gu et al (24)	Men and women	2019	0.79 (0.69–0.90)	45.06
Saito et al (10)	Men	2017	0.72 (0.53–0.98)	8.42
Saito et al (10)	Women	2017	0.73 (0.53–1.01)	7.65
Tohidi et al (17)	Men and women	2017	1.25 (0.48–3.29)	0.86
Zhang et al (11)	Men	2012	1.05 (0.77–1.42)	8.49
Zhang et al (11)	Women	2012	0.55 (0.40–0.76)	7.72
Kurth et al (49)	Women	2007	0.82 (0.55–1.23)	4.91
Soyama et al (7)	Men and women	2003	0.34 (0.14–0.86)	0.97
Leppala et al (51)	Men	1999	0.59 (0.45–0.77)	11.03
Overall^c^	—	—	0.75 (0.68–0.82)	100.0
**Intracerebral hemorrhage**				
Watanabe et al (23)	Men and women	2020	0.53 (0.25–1.14)	6.05
Rist et al (40)	Women	2019	0.98 (0.45–2.13)	5.76
Saito et al (10)	Men	2017	0.81 (0.52–1.28)	17.16
Saito et al (10)	Women	2017	1.72 (1.08–2.74)	16.06
Zhang et al (11)	Men	2012	0.98 (0.52–1.86)	8.57
Zhang et al (11)	Women	2012	2.14 (0.91–5.05)	4.74
Wieberdink et al (43)	Men and women	2011	1.29 (0.48–3.45)	3.58
Noda et al (46)	Men and women	2009	0.98 (0.62–1.53)	17.06
Sturgeon et al (48)	Men and women	2007	1.39 (0.62–2.25)	15.05
Leppala et al (51)	Men	1999	1.33 (0.62–2.85)	5.98
Overall^d^	—	—	1.13 (0.93–1.36)	100.0
**Subarachnoid hemorrhage**				
Watanabe et al (23)	Men and women	2020	0.64 (0.27–1.55)	13.18
Rist et al (40)	Women	2019	1.01 (0.33–3.08)	8.07
Saito et al (10)	Men	2017	1.23 (0.47–3.24)	10.80
Saito et al (10)	Women	2017	0.73 (0.40–1.34)	27.55
Zhang et al (11)	Men	2012	0.56 (0.25–1.25)	15.55
Zhang et al (11)	Women	2012	1.27 (0.50–3.28)	11.38
Leppala et al (51)	Men	1999	0.26 (0.11–0.62)	13.47
Overall^e^	—	—	0.69 (0.50–0.95)	100.0

Abbreviation: —, not applicable.

a Weight = the proportion of the result of each article in the summary results.

b *I* ^2^ = 46.4%; *P* = .05.

c *I* ^2^ = 44.3%; P = 0.06.

d *I* ^2^ = 29.9%; P = 0.17.

e *I* ^2^ = 30.7%; *P* = 0.19.

**Table 3:** Relative Risk For Stroke And Stroke Subtypes in Relation to High-Density Lipoprotein Cholesterol Levels, Systematic Review and Meta-Analysis of Prospective Cohort Studies

Study	Sex	Year	Relative Risk (95% CI)^a^	Weight (%)^b^
**Total stroke**				
Watanabe et al (23)	Men and women	2020	0.69 (0.50–0.95)	6.12
Zhang et al (22)	Men and women	2019	0.81 (0.67–0.96)	19.49
Liu et al (21)	Men	2019	0.86 (0.62–1.19)	5.93
Liu et al (21)	Women	2019	1.19 (0.73–1.94)	2.64
Saito et al (10)	Men	2017	0.75 (0.58–0.97)	9.38
Saito et al (10)	Women	2017	0.92 (0.71–1.19)	9.34
Harandi et al (18)	Men and women	2016	0.92 (0.63–1.34)	4.43
Glasseret al (19)	Men and women	2016	0.93 (0.78–1.10)	21.24
Hirata et al (20)	Men and women	2016	0.86 (0.38–1.93)	0.95
Reina et al (9)	Men and women	2015	0.56 (0.31–0.99)	1.90
Tohidi et al (17)	Men and women	2013	1.11 (0.44–2.78)	0.74
Zhang et al (11)	Men	2012	1.01 (0.66–1.53)	3.60
Zhang et al (11)	Women	2012	0.47 (0.30–0.74)	3.21
Hamer et al (16)	Men and women	2011	1.13 (0.75–1.68)	3.88
Curb et al (8)	Men	2004	0.20 (0.06–0.72)	0.39
Soyama et al (7)	Men and women	2003	0.48 (0.25–0.93)	1.46
Simons et al (6)	Men and women	2001	0.63 (0.42–0.95)	3.83
Wannamethee et al (5)	Men	2000	0.48 (0.25–0.93)	1.46
Overall^c^	—	—	0.82 (0.76–0.89)	100.0
**Ischemic stroke**				
Watanabe et al (23)	Men and women	2020	0.76 (0.51–1.12)	3.55
Gu et al (24)	Men and women	2019	0.75 (0.67–0.83)	10.89
Liu et al (21)	Men	2019	0.83 (0.57–1.21)	3.74
Liu et al (21)	Women	2019	1.14 (0.67–1.93)	2.21
Saito et al (10)	Men	2017	0.68 (0.49–1.01)	4.48
Saito et al (10)	Women	2017	0.70 (0.49–1.01)	3.98
Anne et al (41)	Men and women	2017	0.78 (0.66–0.92)	8.80
Glasser et al (19)	Men and women	2016	0.88 (0.72–1.07)	7.65
Hirata et al (20)	Men and women	2016	1.15 (0.65–2.04)	1.94
Pikula et al (42)	Men and women	2015	0.51 (0.37–0.70)	4.71
Tohidi et al (17)	Men and women	2013	1.44 (0.54–3.84)	0.73
Zhang et al (11)	Men	2012	1.02 (0.62–1.66)	2.47
Zhang et al (11)	Women	2010	0.35 (0.21–0.59)	2.29
Simons et al (45)	Men and women	2010	1.10 (0.65–1.87)	2.21
Willey et al (44)	Men and women	2009	1.08 (0.67–1.66)	2.80
Holme et al (47)	Men	2009	0.79 (0.71–0.86)	11.26
Holme et al (47)	Women	2009	0.67 (0.59–0.73)	10.91
Kurth et al (49)	Women	2007	0.83 (0.53–1.30)	2.90
Psaty et al (50)	Men and women	2004	0.81 (0.60–1.10)	4.90
Soyama et al (7)	Men and women	2003	0.45 (0.21–1.00)	1.09
Leppala et al (51)	Men	1999	0.54 (0.39–0.75)	4.49
Tanne et al (52)	Men	1997	0.55 (0.31–0.93)	2.04
Overall^d^	—	—	0.75 (0.69–0.82)	100.0
**Intracerebral hemorrhage**				
Watanabe et al (23)	Men and women	2020	0.59 (0.28–1.21)	4.72
Rist et al (40)	Women	2019	0.99 (0.47–2.09)	4.53
Saito et al (10)	Men	2017	0.79 (0.49–1.28)	10.91
Saito et al (10)	Women	2017	1.69 (1.05–2.73)	11.01
Zhang et al (11)	Men	2012	1.13 (0.41–3.07)	2.51
Zhang et al (11)	Women	2012	2.64 (0.67–10.42)	1.34
Wieberdink et al (43)	Men and women	2011	1.16 (0.84–1.61)	23.91
Noda et al (46)	Men and women	2009	1.19 (0.82–1.74)	17.75
Sturgeon et al (48)	Men and women	2007	1.69 (1.17–2.41)	19.23
Leppala et al (51)	Men	1999	1.08 (0.49–2.36)	4.10
Overall^e^	—	—	1.21 (1.04–1.42)	100.0
**Subarachnoid hemorrhage**				
Watanabe et al (23)	Men and women	2020	0.64 (0.28–1.49)	0.06
Rist et al (40)	Women	2019	1.02 (0.35–2.97)	0.04
Saito et al (10)	Men	2017	1.53 (0.53–4.41)	0.04
Saito et al (10)	Women	2017	0.98 (0.96–1.00)	99.79
Zhang et al (11)	Men	2012	0.67 (0.19–2.35)	0.03
Zhang et al (11)	Women	2012	1.09 (0.24–4.91)	0.02
Leppala et al (51)	Men	1999	0.41 (0.14–1.23)	0.03
Overall^f^	—	—	0.98 (0.96–1.00)	100.0

Abbreviation: —, not applicable.

a subtypes per 1-mmol/L increase in high-density lipoprotein cholesterol

b Weights are from random effects analysis.

c *I* ^2^ = 42.9%; *P* = .03.

d *I* ^2^ = 50.1%; *P* = .004.

e *I* ^2^ = 33.4%; *P* = .14.

f *I* ^2^ = 0.0%; *P* = .61.

**Table 4:** Dose–Response Subgroup Analyses of Association Between High-Density Lipoprotein Cholesterol and Risk of Total Stroke and Ischemic Stroke, Systematic Review and Meta-Analysis of Prospective Cohort Studies

Characteristics	Total Stroke				Ischemic Stroke			
	No. of Studies	Relative Risk (95% CI)	*I* ^2^	*P *Value^a^	No. of Studies	Relative Risk (95% CI)	*I* ^2^	*P *Value^a^
**All studies**	18	0.82 (0.76–0.89)	42.9	.03	22	0.75 (0.69–0.82)	50.1	.004
**Sex**								
Men/women	7	0.82 (0.70–0.96)	41.8	.11	8	0.78 (0.68–0.89)	51.0	.046
Men	8	0.79 (0.64–0.99)	31.9	.17	9	0.76 (0.65–0.90)	39.6	.10
Women	6	0.74 (0.52–1.04)	60.0	.03	8	0.71 (0.56–0.88)	49.7	.05
**Region**								
Asian	10	0.81 (0.70–0.95)	28.6	.18	10	0.76 (0.68–0.85)	8.0	.37
Non-Asian	8	0.77 (0.63–0.93)	59.2	.02	12	0.74 (0.66–0.84)	65.8	.001
**Follow-up period**								
<10 years	7	0.92 (0.81–1.05)	44.4	.10	9	0.78 (0.66–0.91)	57.7	.03
≥10 years	11	0.77 (0.70–0.85)	31.3	.15	13	0.74 (0.66–0.82)	47.2	.03
**Publication year**								
≤2010	4	0.53 (0.39–0.72)	4.6	.37	8	0.72 (0.63–0.82)	51.1	.05
>2010	14	0.85 (0.78–0.92)	27.0	.17	14	0.77 (0.68–0.87)	51.5	.01
**Sample size**								
<10,000	8	0.67 (0.55–0.82)	30.4	.19	8	0.79 (0.60–1.05)	59.0	.004
≥10,000	10	0.85 (0.78–0.93)	41.2	.08	14	0.75 (0.68–0.81)	47.9	.02
**Alcohol drinking**								
No	6	0.88 (0.79–0.98)	6.5	.38	8	0.74 (0.66–0.83)	59.1	.02
Yes	12	0.76 (0.68–0.85)	48.0	.03	14	0.76 (0.66–0.88)	47.8	.02
**Education**								
No	13	0.80 (0.71–0.91)	31.8	.12	15	0.74 (0.68–0.81)	34.6	.09
Yes	5	0.74 (0.52–1.05)	71.3	.02	7	0.76 (0.62–0.95)	70.4	.002
**Body mass index**								
No	6	0.89 (0.78–1.02)	29.2	.22	6	0.80 (0.70–0.91)	59.4	.03
Yes	12	0.78 (0.71–0.87)	46.3	.04	16	0.72 (0.64–0.81)	48.6	.02
**Systolic blood pressure**								
No	6	0.82 (0.71–0.94)	45.3	.10	6	0.74 (0.65–0.84)	67.6	.009
Yes	12	0.82 (0.74–0.90)	46.7	.04	16	0.76 (0.67–0.86)	42.3	.04
**Physical activity**								
No	10	0.87 (0.74–1.01)	23.6	.23	12	0.76 (0.68–0.84)	52.9	.02
Yes	8	0.73 (0.61–0.88)	53.7	.04	10	0.74 (0.63–0.88)	51.8	.03
**Lipid lowering medication use**								
No	9	0.69 (0.53–0.91)	59.2	.01	14	0.72 (0.65–0.80)	61.6	.001
Yes	9	0.85 (0.77–0.93)	3.6	.41	8	0.83 (0.73–0.93)	0	.62
**Other lipid profiles parameters**								
No	10	0.84 (0.69–1.03)	55.2	.02	12	0.75 (0.67–0.84)	61.0	.003
Yes	8	0.77 (0.69–0.86)	0	.47	10	0.75 (0.64–0.87)	34.9	.13

a Based on the DerSimonian and Laird random-effects model.

**HDL-C level and risk of IS**. We included 10 studies consisting of a total of 706,482 participants and 19,047 people with stroke in the binary analysis of the association of IS risk with HDL-C level. The pooled RR was 0.75 (95% CI, 0.68–0.82; *I* ^2^ = 44.3%; *P*_heterogeneity_ = .06; Table 2). Another 12 studies provided only a continuous risk estimate, so 22 studies were included in the dose–response analysis of IS risk. The pooled RR for IS was 0.75 (95% CI, 0.69–0.82) per 1-mmol/L increase in HDL-C level, with low heterogeneity (*I* ^2^ = 50.1%; *P*_heterogeneity_ = .004) (Table 3). We found a nonlinear dose–response association between HDL-C level and IS risk (*P*_nonlinearity_ = .13) (Figure). No evidence of heterogeneity was detected between subgroups (Table 4). Subgroup analyses showed a nonsignificant association in studies with a sample size of less than 10,000.

**HDL-C level and risk of ICH**. Ten studies consisting of 246,607 participants overall and 1,467 people with ICH were included in the analysis of HDL-C level and risk of ICH. The summary RR was 1.13 (95% CI, 0.93–1.36; *I* ^2^ = 29.9%; *P*_heterogeneity_ = 0.17) in the binary analysis (Table 2). The pooled results showed that risk of ICH was increased 26% per 1-mmol/L increase in HDL-C level (RR 1.21; 95% CI, 1.04–1.42), with low heterogeneity (*I* ^2^ = 33.4%, *P*_heterogeneity_ = 0.14) (Table 3). We found a linear dose–response association between HDL-C level and risk of ICH (P_nonlinearity_ = 0.28) (Figure). The effect size and direction of the pooled estimates were robust for most subgroups.

**HDL-C level and risk of SAH**. Data from 7 studies that included a total of 127,935 participants of which 551 had SAH provided information on the association between HDL-C level and risk of SAH. The pooled RR was 0.69 (95% CI, 0.50–0.95; *I* ^2^ = 30.7%; *P*_heterogeneity_ = 0.19) (Table 2) in the binary analysis. With a per-1–mmol/L increase in HDL-C level, the pooled RR was 0.98 (95% CI, 0.96–1.00; *I* ^2^ = 0%; *P*_heterogeneity_ = 0.61) (Table 3). Hartung-Knapp-Sidik-Jonkman results showed that risk of SAH was decreased 14% per 1-mmol/L increase in HDL-C level (RR 0.86; 95% CI, 0.75–0.98). We found a linear dose–response association between HDL-C level and risk of SAH (*P*_nonlinearity_ = 0.94) (Figure). The pooled estimates remained relatively stable on subgroup analyses.

**Sensitivity analyses and publication bias**. In sensitivity analyses, the results were robust when excluding one study at a time in the analysis of total stroke, IS, ICH, and SAH. We found no publication bias with Begg’s test for risk of total stroke (*P* = 0.10), IS (*P* = .15), and ICH (*P* = .86), and Egger’s test for risk of total stroke (*P* = .10), IS (*P* = .31), and ICH (*P* = .63). Publication bias was not assessed for the association between HDL-C level and SAH because of limited studies.

## Discussion

We aimed to clarify the association between HDL-C level and risk of total stroke and stroke subtypes and found an inverse linear association between HDL-C level and risk of total stroke and IS. For each 1-mmol/L increase in HDL-C level, the risk of total stroke decreased by 18% and that of IS decreased by 24%. For ICH, we found a positive linear association, with the risk of ICH increased 21% per 1-mmol/L increase in HDL-C level. In addition, we found a marginal inverse linear association between HDL-C level and risk of SAH.

Results of previous reviews and meta-analyses evaluating the association between HDL-C level and total stroke, ICH, and SAH were consistent with our study (14,15). However, previous research suggesting a negative association between HDL-C level and total stroke was based on a review of 8 cohort studies and 3 case-control studies (14). Our review did not report the association between HDL-C level and stroke subtypes because of the limited data on that relationship (14). In the current meta-analysis, we quantitatively evaluated the possible linear or nonlinear association of HDL-C level with total stroke, IS, ICH, and SAH.

We found an inverse linear association between HDL-C level and risk of total stroke. The reduced risk of total stroke may be due to the anti-atherosclerotic effects of HDL-C (42). The oxidation of LDL is thought to play an important role in the development of atherogenesis. HDL is a powerful antioxidant that exists in the subintimal space of the artery at a concentration 20 times greater than that of LDL and thus plays an important role in preventing atherosclerosis by inhibiting LDL oxidation in the artery wall (53). Additionally, HDL-C may play a central role in the reverse transport of cholesterol, thereby preventing the accumulation of excess cholesterol in peripheral tissues and the processes that initiate atherogenesis (54). However, subgroup analyses by sex showed significantly decreased risk of total stroke in men but not in women. The reason behind such inference remains unknown, and future experimental studies are needed to explore the potential mechanism.

Among the 22 studies included for the association between HDL-C level and IS risk in the current meta-analysis (7,10,11,17,19–21,23,24,41,42,44,45,47,49–52,42,43,45,46,48,50–53), 16 showed an inverse association (7,10,11,19,20,23,24,41,42,47,49–52), 10 of which reached a significant level (7,10,11,24,41,42,47,51) while the remaining 6 showed no statistical significance (10,19,21,23,49,50). After pooling the 22 studies with a larger sample size, we observed a significant inverse nonlinear association between HDL-C level and IS. The main cause of IS is the formation of atherosclerotic plaque on the carotid artery wall (55). The anti-atherosclerotic effects and potent anti-inflammatory properties of HDL-C could explain our finding of a significant inverse association between HDL-C level and risk of IS (42). The main protein in HDL-C, apolipoprotein A-1, had a direct protective effect on atherosclerosis in several animal experiments (56,57). Besides, Kotur-Stevuljevic et al suggested that the increase in oxidative stress of HDL in patients after IS contributed to a decrease in the activity of the anti-oxidant enzyme paraoxonase 1 (55). Further research should confirm whether increasing HDL-C level through lifestyle changes or pharmacologic therapies will affect IS risk.

Compared with a previous meta-analysis of HDL-C level and hemorrhagic stroke (15), 5 cohort studies were additionally included in our meta-analysis of the association of HDL-C level and ICH risk. We found a positive linear association of HDL-C level and ICH risk, which agreed with the previous meta-analyses. The possible mechanisms are as follows. First, HDL also has an antithrombotic function. A high HDL-C level can increase the risk of ICH by promoting fibrinolysis (10), which was found to be associated with the inhibition of coagulation cascade and the stimulation of blood clot fibrinolysis (58). In addition, HDL attenuates platelet function by stimulating endothelial cells to produce nitric oxide and prostacyclin (58,59).

Results of a previous meta-analysis reported a significant positive association between HDL-C level and SAH based on 2 cohort studies (15). Five cohort studies were additionally included in our meta-analysis of HDL-C level and SAH risk. We found a marginal inverse linear association between HDL-C level and SAH risk. More large-sample cohort studies are needed to firmly establish this association.

Our meta-analysis has several strengths. To our knowledge, this is the first meta-analysis to systematically examine the association between HDL-C level and risk of major stroke subtypes by using both binary and dose–response analyses. Also, all included studies had a prospective design, large sample size, and long follow-up. In addition, the high mean NOS score, 8.24, indicated a relatively high quality of the articles included.

Our meta-analysis also had several limitations. First, IS is a mixed term, including lacunar infarction, large-artery occlusive infarction, and embolic infarction. Only 1 study explored the distinction between IS subtypes, so we could not explore the association between HDL-C level and each IS subtype (10). Second, most included studies did not exclude participants using medication, which may have confounded the association of HDL-C level with risk of total stroke and stroke subtypes. Third, HDL-C level was measured only at baseline, so we could not consider the effect of HDL-C changes during follow-up. Finally, all included studies were observational, and we need further analyses based on randomized clinical trials for assessing the causality of HDL-C level on stroke.

The effects of HDL cholesterol levels on stroke risk vary by type of stroke. A high HDL-C level was associated with reduced risk of total stroke and IS, but an increased risk of ICH. Reasonable control of HDL-C level will prevent and control incident stroke. However, because the HDL particle is so complex, we do not know whether the particle size, number, HDL-C content, or functionality is the best marker of stroke risk. Future studies with information on potential mechanisms are needed.
